# Phytochelatin database: a resource for phytochelatin complexes of nutritional and environmental metals

**DOI:** 10.1093/database/baz083

**Published:** 2019-07-02

**Authors:** Kristine K Dennis, Karan Uppal, Ken H Liu, Chunyu Ma, Bill Liang, Young-Mi Go, Dean P Jones

**Affiliations:** 1 Nutrition and Health Sciences, Laney Graduate School, Emory University, Atlanta, GA, USA; 2 Division of Pulmonary, Allergy, and Critical Care Medicine, Department of Medicine, Emory University, Atlanta, GA, USA

## Abstract

Phytochelatins (PyCs) are a diverse set of plant compounds that chelate metals, protect against metal toxicity and function in metal homeostasis. PyCs are present in plants consumed as food by humans and could, in principle, impact absorption and utilization of essential and toxic metals such as selenium and cadmium, respectively. PyCs vary in terminal amino acid composition and chain length, exist in multiple oxidation states and reversibly bind multiple metals; consequently, PyCs include a large set of possible structures. Although individual PyC-metal complexes have been studied, no resource exists to characterize the diversity of PyCs and PyC-metal complexes. We used the scientific literature to develop a database of elemental formulas for polymer forms varying in chain length from 2 to 11 glutamyl-cysteine repeats. Using elemental formulas, we calculated monoisotopic masses using the most abundant isotopes of each element and calculated masses for complexes with 13 metals of nutritional and toxicological significance. The resulting phytochelatin database (PyCDB) contains 46 260 unique elemental formulas for PyC and PyC-metal complexes. The database is available online for download as well as for direct mass queries for mass spectrometry using an accurate mass annotation tool for user-selected PyC types, metals and adducts of interest. We performed studies of a commonly consumed food—onion—to validate the database and test utility of the tool. Onion samples were analyzed using ultra-high resolution mass spectrometry-based metabolomics. Mass spectral features were annotated using the PyCDB web tool and the R package, xMSannotator; annotated features were further validated by collision-induced dissociation mass spectrometry. The results establish use and a workflow for PyCDB as a resource for characterization of PyCs and PyC-metal complexes.

## Introduction

Phytochelatins (PyCs) function as key mediators of metal detoxification and homeostasis in plants. PyC-metal complexes protect plants from metal toxicity through chelating heavy metals and metalloids such as cadmium (Cd) and arsenic (As). They also bind required nutrients such as zinc (Zn), selenium (Se) and copper (Cu) (1). Due to their essential role, PyCs are extensively studied in agriculture and soil bioremediation (2–4). However, studies focus on analyzing only specific PyCs and a few metals of interest. More comprehensive characterization of PyC-metal complexes would allow greater understanding of metal sequestration and management in plants and additionally, a role for PyCs in metal bioavailability and toxicity in humans and other animal species consuming PyC-containing foods.

PyCs are glutathione (GSH)-derived polypeptides that are formed enzymatically by dipeptidyl transfer of a donor γ-glutamyl-cysteine (γ-Glu-Cys) to GSH or related peptide. The first PyC form identified was (γ-Glu-Cys)_n_-Gly (n = 2–11) (5). Other forms exist in which glycine (Gly) can be substituted with β-alanine (β-Ala), Ala, glutamine (Gln), serine (Ser) or glutamate (Glu) or with no additional amino acid (4,6–8). Because of their high thiol (-SH) content due to Cys residues, PyCs have strong metal-binding abilities with increasing metal capacity with increasing PyC size (3,9).

PyC synthesis is increased in response to metal exposure, allowing plants to bind and mediate risk of heavy metal toxicity. PyCs have been observed in a wide range of plant species (1), accumulating in different tissues depending on the plant (10). For example, in rice (*Oryza sativa*) seedlings exposed to a high dose of Cd, PyC_2_-Gly was at the highest concentration in leaves followed by roots and shoots (6). In a different study of wild basil (*Clinopodium vulgare*) grown with excess Cd, the roots had 4-fold and 10-fold higher concentrations of PyC_2_-Gly compared to leaves and shoots, respectively (11). PyC concentration and lengths also vary with exposure to different metal types and concentrations (11). Functionally, within plants, PyC-metal complexes are not only sequestered in the vacuoles of plant cells but also occur at lower proportions in cytosol and phloem sap (1,12). However, comprehensive characterization of types, concentration and tissue-location of PyCs and PyC-metal complexes has not been completed across common plants grown for human consumption.

**Figure 1:**
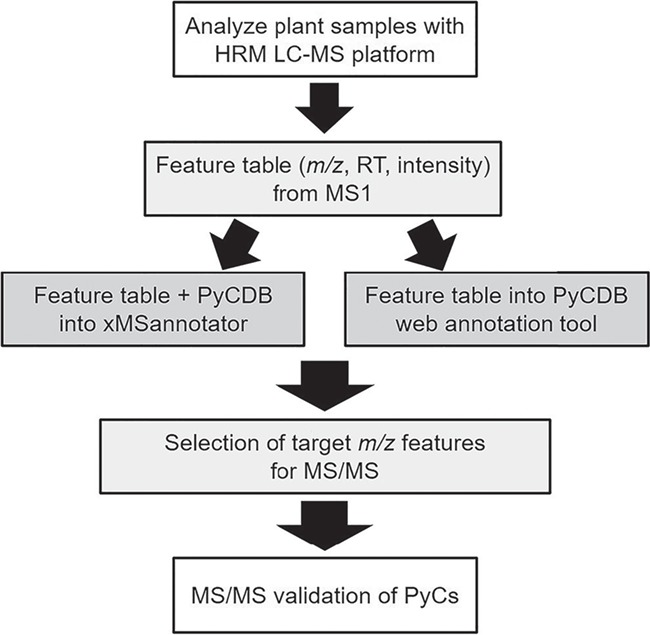
HRM workflow for PyC detection and validation. Using liquid chromatography with ultra-high resolution mass spectrometers followed by the application of data extraction algorithms, broad characterization of the metabolites (mass-to-charge, *m/z*; retention time, RT; and relative intensity) can be obtained. The mass spectral feature table is then searched against the database of compounds using matching criteria such as a retention time window and maximum allowable parts per million differences. Annotated features of interest are then targeted for validation by collision-induced dissociation using MS/MS.

With the development of high-resolution metabolomics (HRM) (Figure 1) (13–16), characterization of multiple forms of metal-free PyC and PyC-metal complexes within a single analysis is possible. However, no database exists to allow characterization of PyCs in plants and food. Here we create a phytochelatin database (PyCDB) for use with metabolomics that contains elemental compositions and monoisotopic masses for a wide range of probable and experimentally detected PyC and PyC-metal complexes. We also provide the PyCDB through an accompanying web-based metabolite annotation tool. We include a metabolomics analysis of a common plant food—onion—as an example for identifying PyCs using the PyCDB in a HRM workflow. Additionally, we provide validation of PyC and PyC-metal complexes included in the PyCDB using collision-induced dissociation mass spectrometry. This database provides a resource for research of PyC and PyC-metal complexes in studies of metals with agricultural, nutritional and toxicological significance.

## Materials and Methods

### Selection of PyCs

Many metals bind with high affinity to thiols (-SH) in Cys residues, and thousands of potential variations of PyC-metal complexes are possible due to the variation in PyC lengths and terminal amino acids. In addition, sulfur can exist in different oxidation states (e.g. thiol versus disulfide form), and metals can interact with multiple functional groups in the peptides (e.g. O- and N-containing groups). Because generation of non-biologically relevant structures in a database can introduce an obstacle to understanding biology, we limited the range of predicted structures to those for which evidence indicates existence or likelihood of existence under relevant biologic conditions. Therefore, to define the scope of the database, we started with thiol (-SH) forms of PyCs. As most metals have the greatest binding affinity to the ionized thiolate (-S^−^) form of thiol groups, preferential binding to thiol groups (over O- and N-containing groups) was assumed for this database iteration. At biological pH (near neutral or slightly basic), a proportion of the thiol groups is expected to be in the metal-binding thiolate form, and experimental evidence shows thiolate binding in PyC-metal complexes (17–19). Based upon this, the PyC-metal complex elemental compositions were calculated accordingly, with two protons removed for every divalent (2+) metal ion bound. For monoisotopic mass calculations based on PyC elemental compositions, we used the most abundant isotope for each element. Finally, predicted compounds were based on likely chemical interactions for common metal oxidation states at neutral pH, room temperature and room air. As knowledge of PyC-metal complexes increases, future versions will be updated to include other PyC-metal complexes.

**Figure 2:**
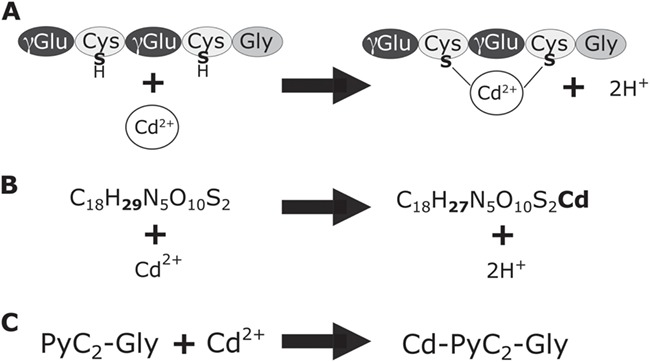
Formation of PyC-metal complexes. **A**. PyCs form complexes with metal ligands. Metal ligands in 2+ oxidation state will bind with the sulfurs of thiol groups on two cysteine residues. **B**. An example of the elemental formulas in PyCDB for the base PyC, phytochelatin2-glycine (PyC_2_-Gly), in metal-bound and unbound forms. **C**. An example of the abbreviated name for the base PyC form, number of repeating peptide units, and metal (if bound).

Using the seven PyC forms (see `Base PyCs’ in Table 1) based upon C-terminal amino acid and repeating γ-Glu-Cys peptide units of 2–11, we calculated base elemental formulas from which predicted forms of PyC-metal complexes were generated (1) (Figure 2). As (γ-Glu-Cys)_n_-Ala and (γ-Glu-Cys)_n_-β-Ala are identical in elemental composition and monoisotopic mass, duplicate elemental compositions were not included. Disulfide (S-S) bonds are formed in molecules with two or more Cys under oxidizing conditions. Disulfide bonds form during food storage and preparation (20). Additionally, disulfide bonds will form during sample preparation unless specific anaerobic conditions or reducing agents are used. Disulfide PyC forms likely also exist *in vivo* due to normal reduction-oxidation reactions occurring as part of cellular signaling and metabolic processes. Single disulfide forms for PyC_2_ to PyC_11_ were calculated by subtracting 2H from the elemental formulas, accounting for the two protons lost from thiol groups during disulfide bond formation. For each additional disulfide, an additional 2H were subtracted. For metal-bound forms, an additional two Cys (-SH groups) will be required for binding of a metal in a 2+ oxidation state. For example, a two-disulfide form can only occur in PyC_4_ or longer, and 2+ metal (Me^2+^)-binding can only occur with a two-disulfide form in PyC_6_ or longer. Up to five disulfide forms were included corresponding to known PyC lengths (Table 1).

**Table 1:** PyC structures vary by terminal amino acid, number of repeating peptide units (n = 2–11) and number of disulfide bonds (m = 1–5)

**Base PyCs**	**Elemental formula (e.g. n = 2)**	**Disulfide form**
(γ-Glu-Cys)_n_-Gly	C_18_H_29_N_5_O_10_S_2_	(S-S)_m_(γ-Glu-Cys)_n_-Gly
(γ-Glu-Cys)_n_-β-Ala	C_19_H_31_N_5_O_10_S_2_	(S-S)_m_(γ-Glu-Cys)_n_-β-Ala
(γ-Glu-Cys)_n_-Ala	C_19_H_31_N_5_O_10_S_2_	(S-S)_m_(γ-Glu-Cys)_n_-Ala
(γ-Glu-Cys)_n_	C_16_H_26_N_4_O_9_S_2_	(S-S)_m_(γ-Glu-Cys)_n_
(γ-Glu-Cys)_n_-Gln	C_21_H_34_N_6_O_11_S_2_	(S-S)_m_(γ-Glu-Cys)_n_-Gln
(γ-Glu-Cys)_n_-Ser	C_19_H_31_N_5_O_11_S_2_	(S-S)_m_(γ-Glu-Cys)_n_-Ser
(γ-Glu-Cys)_n_-Glu	C_21_H_33_N_5_O_12_S_2_	(S-S)_m_(γ-Glu-Cys)_n_-Glu

PyC-metal complexes were calculated using the elemental formulas described above as base units for construction of other forms. If two thiols were not available for binding, the PyC-metal complex was not included.

### Selection of metals

Database metals include selected metals of either nutritional or toxicological importance. Other factors considered for metal selection included the common oxidation states, most abundant isotopes and expected Lewis acid-base chemistry. The Lewis acid-base chemistry influences the ability of metals to form stable complexes. This relates to whether the metal ion is a hard acid or soft acid. Soft acids have greater tendency to form stable complexes with thiolates (-S^−^) (21,22). Of note but beyond the scope of the current discussion, the molecular environment of the thiol group will also impact the affinity to which it binds the metal. The molecular environment will be influenced by factors such as the biological matrix and sample preparation. For example, the binding affinity of metals to the thiolate will change depending on pH. These factors must be considered when designing experiments and interpreting results for studies of PyC-metal complexes.

Many of the soft metals of nutritional and toxicological interest have a common oxidation state of 2+: cadmium (Cd), cobalt (Co), copper (Cu), iron (Fe), lead (Pb), manganese (Mn), mercury (Hg), nickel (Ni) and zinc (Zn). Some have common oxidation states of 1+: Cu, Hg and silver (Ag). Initial calculations were made for the 2+ ions of the most abundant isotope, including ^114^Cd, ^59^Co, ^63^Cu, ^56^Fe, ^208^Pb, ^55^Mn, ^202^Hg, ^58^Ni and ^64^Zn, for the 1+ ion (^107^Ag), and for negatively charged (2-) forms of selenium (^80^Se). This includes five nutritionally important minerals (Fe, Cu, Mn, Se and Zn) and eight metals of environmental health concern (Cd, Co, Pb, Mn, Hg, Ni, Ag and Se) (Supplementary Information, Figure S-2). Some metals, such as Se and Mn, are both nutritional and toxicological metals due to tolerable upper intake limits (i.e. Mn, adults, 11 mg/day; Se, adults, 400 μg/day) being relatively low compared to some exposure levels in humans. Although calcium (^40^Ca, 2+) and magnesium (^24^Mg, 2+) are hard acids and may not bind effectively to thiols, they are abundant in plants and could be present as PyC complexes (22). These Ca and Mg forms were included in the current database and additional forms for potassium (^39^K, 1+) and sodium (^23^Na, 1+) could be included in future database iterations.

### PyC-metal complexes

PyC-metal complexes including one or two metals for 2+ ions and Se (2-) were calculated by subtracting 2H and 4H, respectively, from base elemental formulas to account for the loss of thiol protons during metal binding. For selenium, the calculations were based upon experimental results demonstrating that selenite reacts with two PyC_2_ to create a bound PyC_2_-Se and an oxidized (S-S)PyC_2_. The product was shown to be the selenotrisulfide, -S-Se-S- (23), where the selenium of selenite was reduced to the formal 2+ oxidation state in binding to two thiolates. Ag (1+) one-metal and two-metal forms were calculated by subtracting 1H per metal addition. For two metal (2+) bound forms, at least four thiols would be needed. Therefore, calculations for two metal (2+) forms were completed for PyC_4_ and larger, one-disulfide forms for PyC_6_ and larger, two-disulfide forms for PyC_8_ and larger and three-disulfide forms for PyC_10_ and PyC_11_. Two metal complexes were calculated for all possible combinations of included metals, recognizing that the precise structure of the complexes cannot be predicted based upon these calculations. A larger number of metals and metal combinations could bind to longer chain lengths (up to 5 in PyC_10_ or PyC_11_ forms). However, these were not included in this PyCDB version because of the large number of combinations and limited evidence for such complex forms.

For all two-metal PyC-metal complexes, bridging sulfurs, as occur in iron-sulfur clusters, are possible for most of the metals included in this database iteration (24). We referred to these as sulfido and disulfido forms. The elemental formulas were calculated by adding either one or two sulfurs to elemental formulas for two metal complexes. As selenide may form similar complexes, these were calculated by addition of one selenium to the elemental compositions, referred to as the selenido form. Diselenido forms will be included in future database versions if disulfido forms are found.

### Calculation of monoisotopic masses

Monoisotopic masses were calculated using the elemental formulas of the predicted compounds. Monoisotopic mass values were generated for each PyC and PyC-metal complex using a modification of the R package, OrgMassSpecR. This R package allows for automated calculations of monoisotopic masses using elemental formulas. Modifications were made to the functions *ListFormula* and *MonoisotopicMass* to include all elements of interest. Monoisotopic mass calculations were completed using the National Institute of Standards and Technology exact masses (rounded to eight decimal places) for the most abundant isotopes (25).

### PyCDB web interface

The PyCDB web tool was developed using the shiny package, shinyBS package and DT package in R. The web interface is maintained in the shiny server. Users can enter their experimental masses directly in a text box or upload them with `.csv’ format or `.txt’ format to perform accurate mass matching within the PyCDB web tool. Using the *get_mz_by_monoisotopicmass* function in xMSannotator, the mass-to-charge ratio (*m/z*) for each PyC and PyC-metal complex for adducts of interest were calculated by adding the mass of the respective adduct (e.g. M + H, M + 2H) and dividing by the charge state (*z* = 1,2,3) of the adduct (26). For accurate mass matching with the web tool, there is the option to include annotation only for elemental formulas that meet the nitrogen (N), oxygen (O), phosphorus (P) and sulfur (S) to carbon (NOPS) ratio check (27). The NOPS check allows filtering out elemental formulas that do not include the most common ratios of N, O, P and S atoms to carbon. The user-defined input parameters can be specified and used by the back-end R function to find PyC/PyC-metal matches. Once the processing is complete, a table of PyC/PyC-metal matches is available in the web tool or for download as a .csv file.

### Using the PyCDB locally

The user has the option of using the full database locally with existing tools or R packages, such as xMSannotator (26). With the xMSannotator *multilevelannotation* function, annotation criteria are available in addition to those available via the PyCDB web interface. The output of *multilevelannotation* includes a confidence level (i.e. none, low, medium, high) for annotated metabolites. Additional criteria include retention time clustering, hydrogen/carbon ratio checks, adduct requirements specified by the user for high confidence scores (e.g. M + H for high confidence), and abundance ratio checks for isotopes and multiply charged adducts.

## Results

### PyCDB content

The current database includes 46 260 unique elemental formulas for 240 PyC and 46 020 PyC-metal complexes. Information available for each complex includes the molecular formula, monoisotopic mass, isotope, PyC type (see Table 1), number of repeat units (e.g. PyC_2_ to PyC_11_) and information on bound metals (including type and number). With increasing PyC length, the number of compounds in the database increases due to the higher number of metal-binding sites in longer peptides (Supplementary Information, Figure S-2).

### PyCDB website implementation

**Figure 3:**
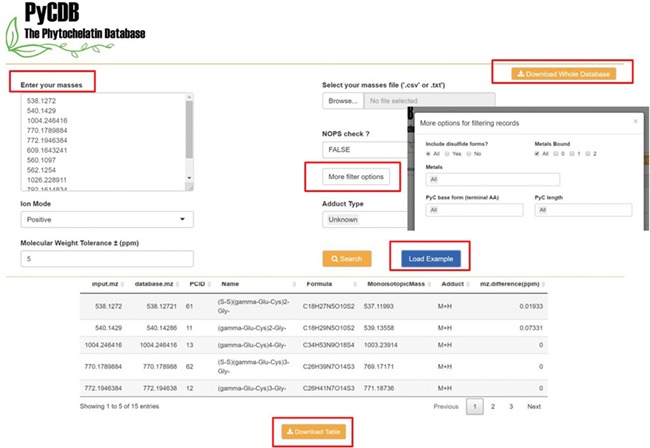
Screenshots of the web version of the PyCDB. Query masses of interest can be entered manually or uploaded from a .csv or .txt file. Screenshot of an example search using the `Load Example’ button with the default search settings and the output shown below. The output can be reviewed on the webpage or downloaded as a .csv file for future use. The webpage also provides an option to `Download Whole Database’ for use with other annotation tools such as the R package, xMSannotator.

The database is available in a user-friendly form from https://kuppal.shinyapps.io/pycdb/. As seen in Figure 3, users can select specific search criteria after uploading their experimental masses. Search options include selecting a subset of the 13 metals, adducts of interest and specific PyC lengths or types (i.e. terminal amino acid). The `NOPS check’ option can help users further filter less likely masses based on the ratio of NOPS atoms to carbon in the chemical formula (27). The user can also define the mass error in parts per million (ppm; e.g. 5 ppm) to perform the accurate mass search. As described above, the user-defined input parameters are used by the back-end R function to find PyC matches. The full database is also available for download at https://s3.amazonaws.com/phytochelatindatabase/full_version_PyCDB_20180821.csv, which can be used with other annotation tools like xMSannotator for users interested in additional annotation criteria (26).

### Example: PyC detection in onions

Samples from eight onions were analyzed using HRM. Detected metabolites were defined by accurate mass (*m/z*), retention time (seconds) and intensity profiles of mass spectral features. Details of the sample preparation and analysis are described in the Supplementary Information. A total of 19 270 features were detected on the C18 column. The feature table was analyzed in two ways, targeting a subset of the database (Supplementary Information). First, the feature table was annotated using xMSannotator with the custom database option. The xMSannotator code used for the analysis is provided in the Supplementary Information. A total of 628 features were annotated using the PyCDB subset. The feature table was also analyzed in the PyCDB web tool using the same parameters as xMSannotator but without the NOPS check. The PyCDB web tool uses a simple annotation function, which does not provide confidence scores. This resulted in 845 annotated features.

Using selection criteria based on high confidence matches from xMSannotator and high intensity features, features were selected for further validation with collision-induced dissociation mass spectrometry (MS/MS). The feature *m/z* 538.1270 was matched to (S-S)PyC_2_-Gly (C18H27N5O10S2) [M + H]. MS/MS of this *m/z* target was completed to confirm the identity (see Figure S-1). Many annotated PyC-metal complexes from the onion data were at too-low intensity for collision-induced dissociation analysis. PyC-metal complexes formed using chemical standards and selected metals (see Supplementary Information) showed that experimental masses were consistent with predicted masses. Additional details of metabolite validation and PyC-metal complex collision-induced dissociation are provided in the Supplementary Information (Figure S-1 and Figure S-3 to S-8). Examples are provided along with literature data for PyCs and PyC-metal complexes in Table 2 (6,18,19,28–30).

**Table 2:** Comparisons of predicted masses in database with experimental masses of PyCs identified by mass spectrometry

PyC	Predicted mass (*m/z*)	Experimental mass (*m/z*)	Mass deviation (ppm)	Adduct
Unbound PyCs				
(S-S)PyC_2_-Gly^a^	538.1272	538.1254^a^	3.3	M + H
PyC_2_-Gly^a^^,^^b^	540.1429	540.1437^a^, 540.1^(18)^, 540.1430^(6)^, 540.0^(28)^	1.5, −, 0.1, −	M + H
PyC_3_-Gly^a^^,^^b^	772.1946	772.1955^a^, 772.2^(18)^, 772.1948^(6)^, 772.0^(28)^	1.2, −, 0.3, −	M + H
PyC_4_-Gly^a^^,^^b^	1004.2464	1004.2436^a^, 1004.2^(18)^, 1004.2458^(6)^, 1004.0^(28)^	2.8, −, 0.6, −	M + H
PyC_5_-Gly^b^	1236.2982	1236.2^(18)^	-	M + H
PyC_6_-Gly^b^	1468.35	1468.2^(18)^	-	M + H
PyC_2_-Ser^b^	570.1534	570.1538^(6)^	0.7	M + H
PyC_3_-Ser^b^	802.2052	802.206^(6)^	1	M + H
PyC_4_-Ser^b^	1034.257	1034.2577^(6)^	0.7	M + H
PyC_2_-Gln^b^	611.18	611.1802^(6)^	0.3	M + H
PyC_3_-Gln^b^	843.2318	843.2328^(6)^	1.2	M + H
PyC_2_-Glu^b^	612.164	612.1648^(6)^	1.3	M + H
PyC_3_-Glu^b^	844.2158	844.2167^(6)^	1.1	M + H
PyC_4_-Glu^b^	1076.2675	1076.2686^(6)^	1	M + H
PyC-metal complexes				
PyC_2_-Gly-Hg^b^	740.0979	740^(29)^, 740.1^(30)^	-	M + H
PyC_3_-Gly-Hg^b^	972.1496	972^(29)^, 972.1^(30)^	-	M + H
PyC_4_-Gly-Hg^b^	1202.1858	1202^(29)^, 1202.2^(30)^	-	M + H
PyC_4_-Gly-Hg(2)^b^	1404.1564	1404^(29)^, 1404.2^(30)^	-	M + H
PyC_2_-Gly-Cd^a^	652.0306	652.0346^a^	6.1	M + H
PyC_3_-Gly-Cd^a^^,^^b^^,^^c^	884.0824	884.0710^a^^,^^c^, 884.1^(18)^	12.9, −	M + H
PyC_4_-Gly-Cd^a^^,^^c^	1116.1341	1116.1392^a^^,^^c^, 1116.1^(18)^	4.6, −	M + H
PyC_4_-Gly-Cd(2)^a^	1228.0218	1228.0279^a^	5	M + H
PyC_5_-Gly-Cd^b^	1348.1859	1348.2^(18)^	-	M + H
PyC_2_-Pb^b^	746.1039	746.1034^(19)^	0.7	M + H
PyC_3_-Pb^b^	978.1556	978.1559^(19)^	0.3	M + H
PyC_4_-Pb^b^	1210.2074	1210.1986^(19)^	7.3	M + H
PyC_4_-Pb(2)^b^	1416.1684	1416.1556^(19)^	9	M + H
PyC_2_-Zn^a^^,^^b^^,^^c^	602.0564	602.0560^a^^,^^c^, 602.0544^(19)^	0.7, 3.3	M + H
PyC_3_-Zn^a^^,^^c^	834.1081	834.1136^a^^,^^c^	6.6	M + H
PyC_4_-Zn^a^^,^^c^	1066.1599	1066.1608^a^^,^^c^	0.8	M + H
PyC_4_-Zn(2)^a^^,^^c^	1128.0734	1128.0763^a^^,^^c^	2.6	M + H
PyC_2_-Mn^a^	593.0653	593.0665^a^	2	M + H
PyC_3_-Mn^a^	825.1170	825.1251^a^	9.8	M + H
PyC_4_-Mn^a^	1057.1688	1057.1672^a^	1.5	M + H
PyC_4_-Mn(2)^a^	1110.0912	1110.0913^a^	0.1	M + H

a ^a^Compared with standards; see supplementary information.

b ^b^Previously identified PyC derivative with mass as reported.

c ^c^Data not shown.

## Discussion

### Future directions

The database is open source and can be refined as plant and food metabolomics data are compared to the predicted complexes. Such modifications can include additional metals of nutritional and toxicological significance such as molybdenum and arsenic. Although selenocysteine (Sec) is not common in plants, Sec may form in the PyC in high-selenium conditions and be of interest to explore. Other metals and metal oxidation forms are possible. The complex coordination chemistries of the metals will need to be considered for inclusion. To aid in confirmation of identities, additional methods, such as metal removal or study by ion mobility spectrometry-mass spectrometry, may be needed to address low abundance of the PyC-metal complexes in biologic materials. Although up to two metals were only considered in the current database iteration, PyC binding of up to five metals for longer forms is chemically possible and could be explored. Finally, a future iteration of the PyCDB could include an *in silico* fragmentation tool to account for predicted MS/MS spectra of PyC-metal complexes. Although peptide fragmentation tools are available, predicted fragmentation for PyC-metal complexes will need to consider metal interactions with O- and N-containing carboxy and amino groups as well as sulfido, disulfido and selenido PyC forms included in the database.

In addition to the potential applications of the PyCDB for understanding metal homeostasis in plants and absorption of nutritional and toxicological metals in plant-derived foods, the PyCDB could also be useful for understanding metal-dependent processes such as nutritional immunity. Nutritional immunity is the process by which a host controls access to micronutrients to protect from bacterial infections (31,32), and PyCs could contribute to mechanisms of nutritional immunity due to their metal-binding characteristics. Thus, the PyCDB has the potential to be a useful resource for diverse *in vivo* and *in vitro* investigations of PyCs and PyC-metal complexes.

### Conclusions

The range of potential PyCs and PyC-metal complexes in plants and food products is extensive and diverse. The PyCDB provides a foundational resource for research efforts to characterize PyC profiles. Here we demonstrate PyCs can be detected and validated in the edible portion of a commonly consumed plant food, onion. Additionally, PyCs and PyC-metal complexes formed *in vitro* are detected at the predicted masses in the database. Future database versions can include additional PyC forms and validated compounds. Given the significant role of PyCs in binding metals of toxicological and nutritional significance, this database provides a resource to improve understanding of PyCs in metal homeostasis and metal bioavailability in plants and plant-derived foods consumed by animals.

## Supplementary Material

PyCDB_supplementaryinfo_revisions_4_27_19_clean_baz083

## Notes

**Database URL:** https://kuppal.shinyapps.io/pycdb/
