# Supplementary material for: Phytochelatin database: a resource for phytochelatin complexes of nutritional and environmental metals
Source: Database (Oxford). 2019 Jul 2;2019:baz083. doi: 10.1093/database/baz083 (PMC6606759; doi:10.1093/database/baz083)
Supplement: PyCDB_supplementaryinfo_revisions_4_27_19_clean_baz083 [file pycdb_supplementaryinfo_revisions_4_27_19_clean_baz083.doc]

**Supplementary Information**

Phytochelatin database (PyCDB): A resource for phytochelatin complexes of nutritional and environmental metals

Kristine K. Dennis^1,2^, Karan Uppal^2^, Ken H. Liu^2^, Chunyu Ma^2^, Bill Liang^2^, Young-Mi Go^2^, Dean P. Jones^1,2^

^1^ Nutrition and Health Sciences, Laney Graduate School, Emory University, Atlanta, GA

^2^ Division of Pulmonary, Allergy, and Critical Care Medicine, Department of Medicine, Emory University, Atlanta, GA.

**Abstract**

The supplementary information includes the methods of onion sample preparation and metabolomics analysis, annotation of onion features with xMSannotator, LC-MS/MS validation of PyC_2_-Gly in onions, R script used for annotation with xMSannotator, and MS/MS results for PyC authentic standards (PyC_2_-Gly, PyC_3_-Gly, PyC_4_-Gly) complexed with manganese and cadmium.

**Metabolomics of onions**

Eight onions were selected for analysis. 100mg pieces of each onion were frozen at -80*^o^C*. Metabolites were extracted from onion samples using a Polytron homogenizer in 400µL extraction solution (2:1 acetonitrile to water) and homogenates were centrifuged for 10 minutes at 14,000rpms at 4*^o^C*. Metabolites in the supernatant were collected, loaded into auto-sampler vials, and run on a high resolution LTQ-Velos Orbitrap mass spectrometer (Thermo Fisher). Samples were analyzed in triplicate with a 10µl injection volume on a C18 column in positive electrospray ionization mode using a previously described method (1,2). The data were extracted using the R packages apLCMS and xMSanalyzer to provide a table of detected metabolites (mass spectral features defined by accurate mass (*m/z*), retention time (RT), and intensity profiles) (3,4).

**Annotation of onion data with xMSannotator**

Feature tables were analyzed using xMSannotator and the *multilevelannotation* function with the custom database option as defined in the R script below. Based on the expected concentration and frequency of detected PyC lengths from previous research, a subset of the PyCDB was run with only PyC_2_ to PyC_6_, focusing on forms with one or no metals (5,6). xMSannotator allows accurate mass matching (Level 5 identification according to Schymanski et al. 2014) based on criteria such as correlation analysis, network modularity analysis, RT-based clustering, and mass defect analysis to assign confidence scores (0-3) to the annotations (7,8). Multiple adducts were considered (i.e., M+H, M+Na, M+K, M+2H, M+3H, M+NH_4_) with the M+H adduct required for the highest confidence level to be assigned. Using these parameters, 628 features were annotated using the PyCDB subset defined above.

**Validation of phytochelatin with MS/MS**

MS/MS of *m/z* 538.1270 was completed on the LTQ-Velos Orbitrap. The identification was confirmed via MS/MS in positive mode using collision-induced dissociation at 35V on the LTQ-Velos Orbitrap. Validation of (S-S)PyC_2_-Gly with MS/MS was performed in two ways. First, MS/MS onion spectra were uploaded into MetFrag for matching (9,10). MetFrag links with PubChem, which has three phytochelatins in the database (PyC_2_-Gly, PyC_3_-Gly, PyC_4_-Gly) (11). From MS/MS spectra for *m/z* 538.1270, MetFrag returned a top match for (S-S)PyC_2_-Gly (C18H27N5O10S2). An example of a representative MS/MS spectra can be seen in Figure S-1B. As an authentic standard is available for PyC_2_-Gly, MS/MS analysis was performed on the LTQ-Velos Orbitrap to generate a reference spectra (Figure S-1A). Validation was also performed via matching of fragmentation patterns of the onion spectra (Figure S-1B) with the spectra of the PyC_2_-Gly authentic standard.

**Mass fragmentation analysis of PyC and PyC-metal complex standards**

Analytical standards of PyC_2_-Gly (95% purity),PyC_3_-Gly (95% purity), and PyC_4_-Gly (95% purity) were obtained from CPC Scientific Inc. CdCl_2_ and MnCl_2_ were obtained from Sigma-Aldrich. Individual stock solutions of PyC_2_, PyC_3_, CdCl_2_, and MnCl_2_ were prepared in HPLC-grade water. PyC_2_-Gly, PyC_3_-Gly and PyC_4_-Gly were prepared at 10µM. PyC-metal complex solutions were prepared in water at 10µM:10µM PyC to metal ratios and were analyzed with direct injection mass spectrometry using a LTQ-Velos Orbitrap mass spectrometer (Thermo Fisher).

**R script for annotation of onion metabolites with xMSannotator**

1. Read in PyCDB as custom database file:

PC_full<-read.csv("full_version_PyCDB_20180821.csv")

1. Create subset of PyCDB for query (optional):

PC_full$PCkeep<-ifelse((PC_full$PC.General %in% c("PC2","PC3","PC4","PC5","PC6")) & (PC_full$Metal.Form %in% c("0","1")), 1,0)

PC2_PC6<-subset(PC_full, PC_full$PCkeep ==1)

1. Read in data table:

dataA<-read.table("~/PyC/FoodFiles/onion/C18/C18_Onion_Stage3b_filter.txt", header=TRUE)

1. Specify search parameters

max.mz.diff<-10 #mass search tolerance for DB matching in ppm

max.rt.diff<-10 #retention time tolerance between adducts/isotopes

corthresh<-0.7 #correlation threshold between adducts/isotopes

max_isp=5 #maximum number of isotopes to search for

mass_defect_window=0.01 #mass defect window for isotope search

1. Specify output location

outloc<-"~/PyC/FoodFiles/C18/onion_PyCDBpaper/"

1. Specify number of cores to be used

num_nodes<-4

1. Specify name of database to search against

db_name="Custom"

status=NA

customIDs<-NA

1. Specify number of sets the total database entries should be split into for searches

num_sets<-300

1. Specify ionization mode and adducts for search

mode<-"pos"

queryadductlist=c("M+2H","M+H","M+Na","M+K", "M+3H","M+NH4")

1. Provide name of custom database to be used for annotation

customDB<-PC2_PC6

1. xMSannotator multilevel annotation function

#########################

dataA<-unique(dataA)

print(dim(dataA))

print(format(Sys.time(), "%a %b %d %X %Y"))

system.time(annotres<-multilevelannotation(dataA=dataA,max.mz.diff=max.mz.diff,max.rt.diff=max.rt.diff,cormethod="pearson",num_nodes=num_nodes,queryadductlist=queryadductlist,

mode=mode,outloc=outloc,db_name=db_name, adduct_weights=NA,num_sets=num_sets,allsteps=TRUE,

corthresh=corthresh,NOPS_check=TRUE,customIDs=customIDs,missing.value=NA,deepsplit=2,networktype="unsigned",

minclustsize=10,module.merge.dissimilarity=0.2,filter.by=c("M+H"),biofluid.location=NA,origin=NA,status=status,boostIDs=NA,max_isp=max_isp, customDB=customDB, HMDBselect=NA,mass_defect_window=mass_defect_window,pathwaycheckmode="pm",mass_defect_mode="pos")

)

print(format(Sys.time(), "%a %b %d %X %Y"))


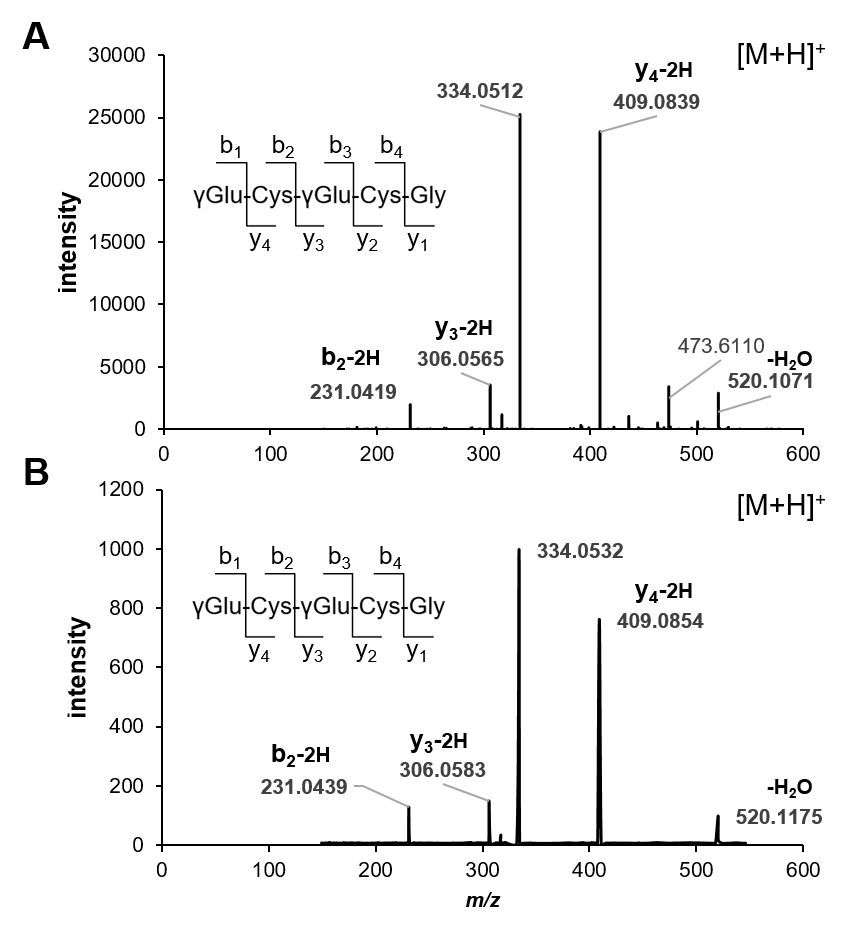


**Figure S-1.** Matching MS/MS spectra of *m/z* 538.1272 [M+H] for PyC_2_-Gly authentic standard and onion samples (representative spectra shown). **A**) Fragmentation pattern for (S-S)PyC_2_-Gly [M+H] from the authentic standard sample using collision-induced dissociation (CID) at 25V **B**) Fragmentation pattern for (S-S)PyC_2_-Gly [M+H] from an onion sample using CID at 35V.

**
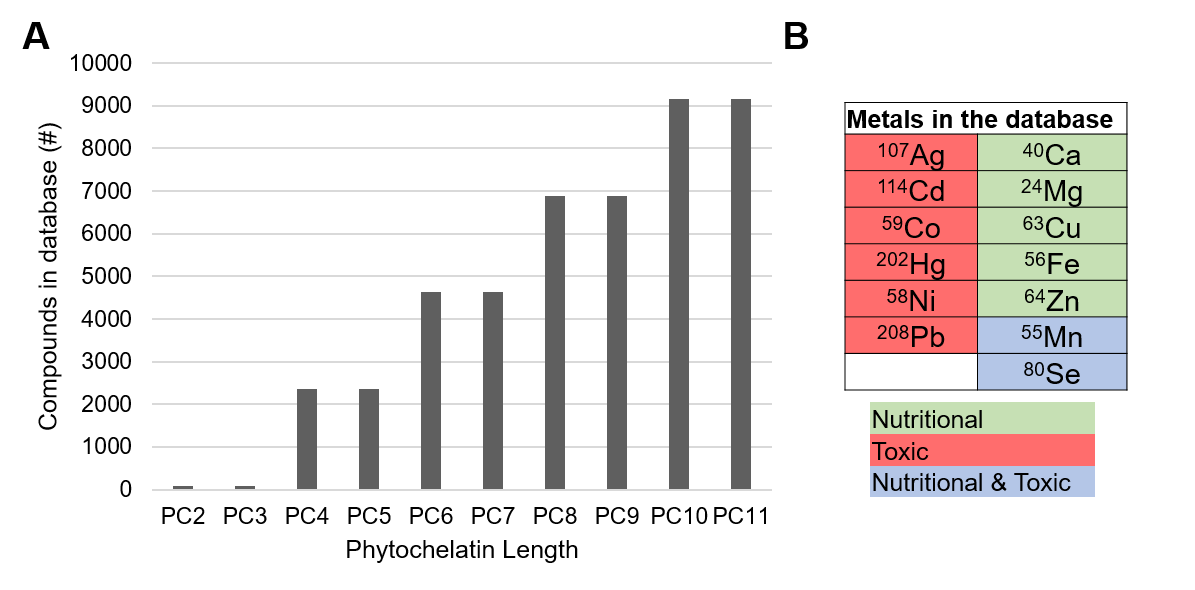
**

**Figure S-2.** **Characteristics of the database.** **A**) Number of compounds in database by phytochelatin length (i.e., repeating peptide units, n=2-11). With increasing phytochelatin length, the number of possible phytochelatins and phytochelatin-metal complexes increases. **B**) The most common isotope of metals of nutritional and toxicological significance are included in the database.


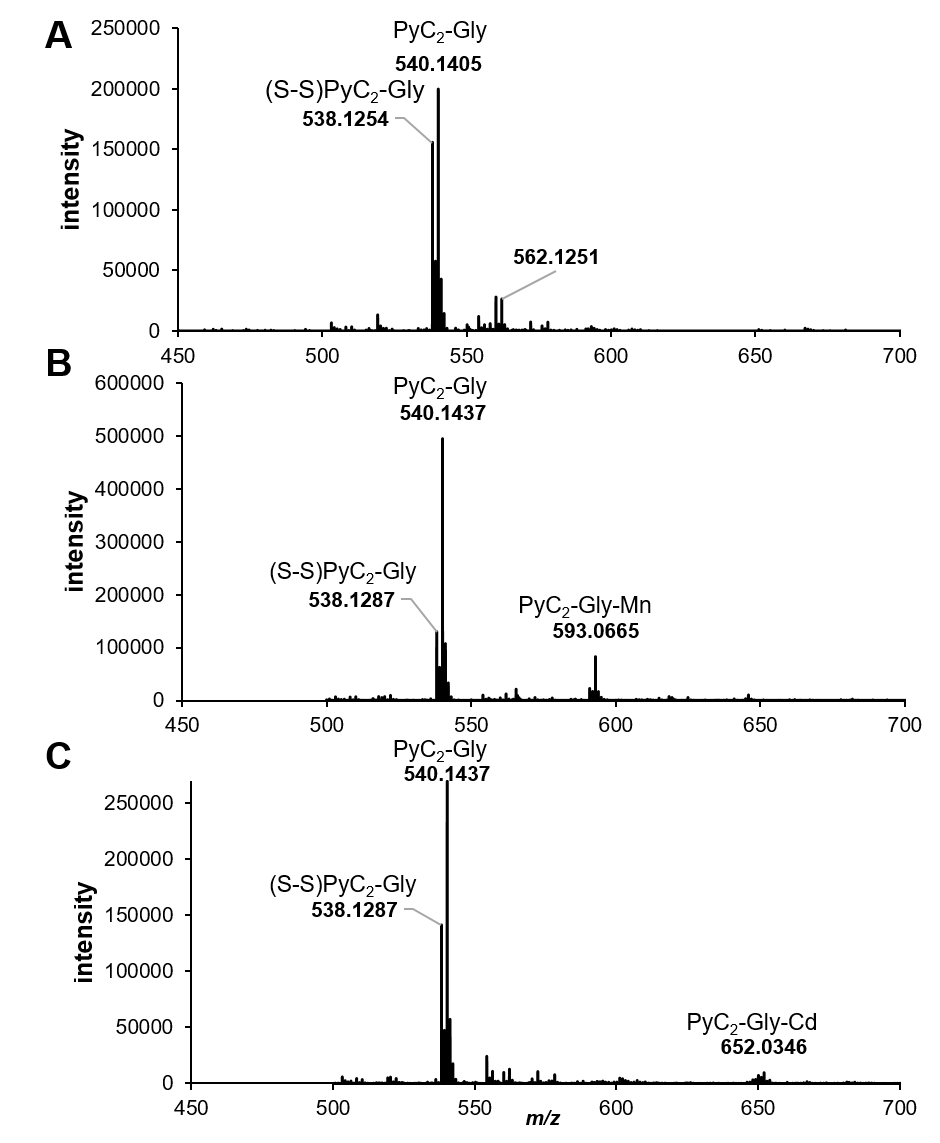


**Figure S-3.** MS1 spectra of PyC_2_-Gly authentic standard alone (**A**) and in combination with equimolar MnCl_2_ (**B**) or CdCl_2_ (**C**). (S-S)PyC_2_-Gly, oxidized form of PyC_2_-Gly; PyC_2_-Gly, reduced form of PyC_2_-Gly; PyC_2_-Gly-Mn, PyC_2_-Gly complexed with Mn^2+^ ion; PyC_2_-Gly-Cd, PyC_2_-Gly complexed with Cd^2+^ ion.


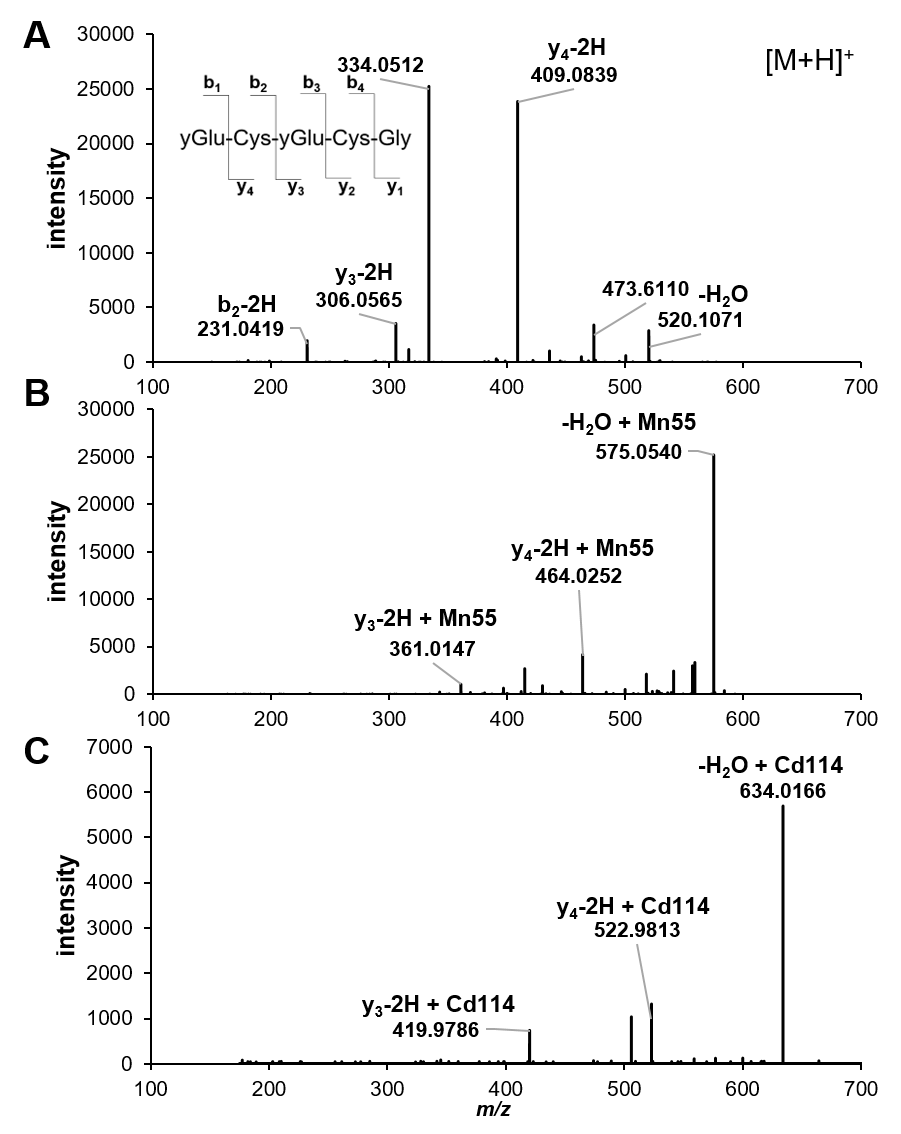


**Figure S-4. MS/MS of (S-S)PyC_2_-Gly, PyC_2_-Gly-Mn, and PyC_2_-Gly-Cd. A**) Fragmentation pattern for (S-S)PyC_2_-Gly [M+H] (*m/z* 538.13) from the authentic standard sample using collision-induced dissociation (CID) at 25V, **B**) for PyC_2_-Gly-Mn [M+H] (*m/z* 593.07) from the authentic standard mixed with equimolar MnCl_2_ using CID25V, and **C**) for PyC_2_-Gly-Cd [M+H] (*m/z* 652.03) from the authentic standard mixed with equimolar CdCl_2_ using CID35V.


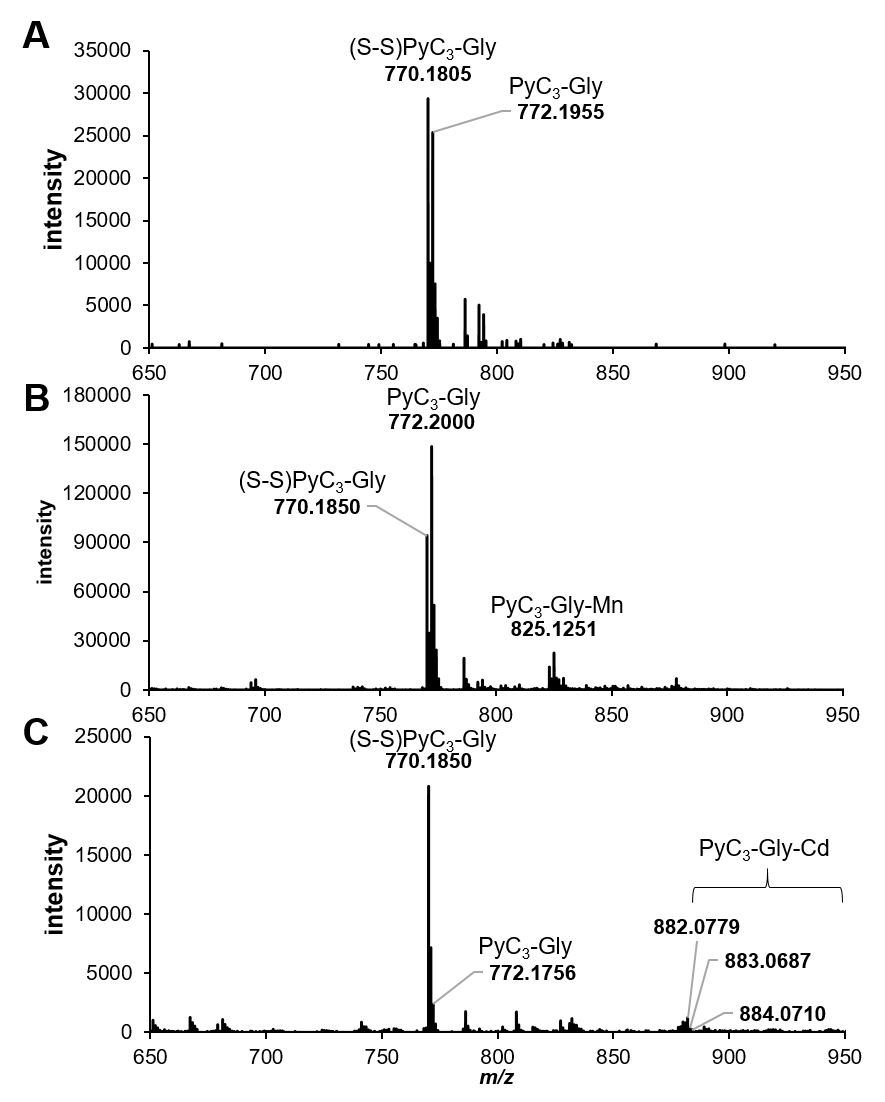


**Figure S-5.** MS1 spectra of PyC_3_-Gly authentic standard alone (**A**) and in combination with equimolar MnCl_2_ (**B**) or CdCl_2_ (**C**). (S-S)PyC_3_-Gly, oxidized form of PyC_3_-Gly; PyC_3_-Gly, reduced form of PyC_3_-Gly; PyC_3_-Gly-Mn, PyC_3_-Gly complexed with Mn^2+^ ion; PyC_3_-Gly-Cd, PyC_3_-Gly complexed with Cd^2+^ ion.


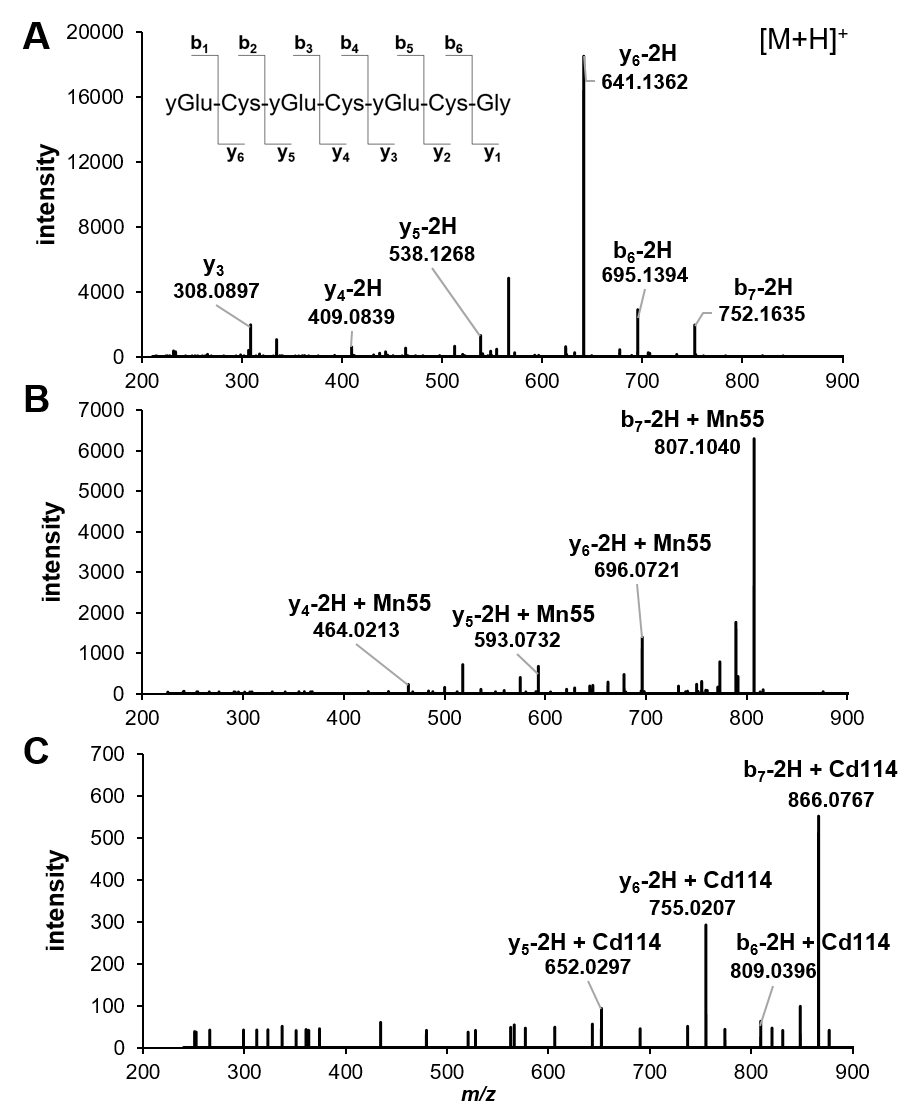


**Figure S-6.** **MS/MS of (S-S)PyC_3_-Gly, PyC_3_-Gly-Mn, and PyC_3_-Gly-Cd. A)** Fragmentation pattern for (S-S)PyC_3_-Gly [M+H] (*m/z* 770.18) from the authentic standard sample, **B)** for PyC_3_-Gly-Mn [M+H] (*m/z* 825.12) from the authentic standard mixed with equimolar MnCl_2_, and **C)** for PyC_3_-Gly-Cd [M+H] (*m/z* 884.09) from the authentic standard mixed with equimolar CdCl_2_ using collision-induced dissociation (CID) at 25V.


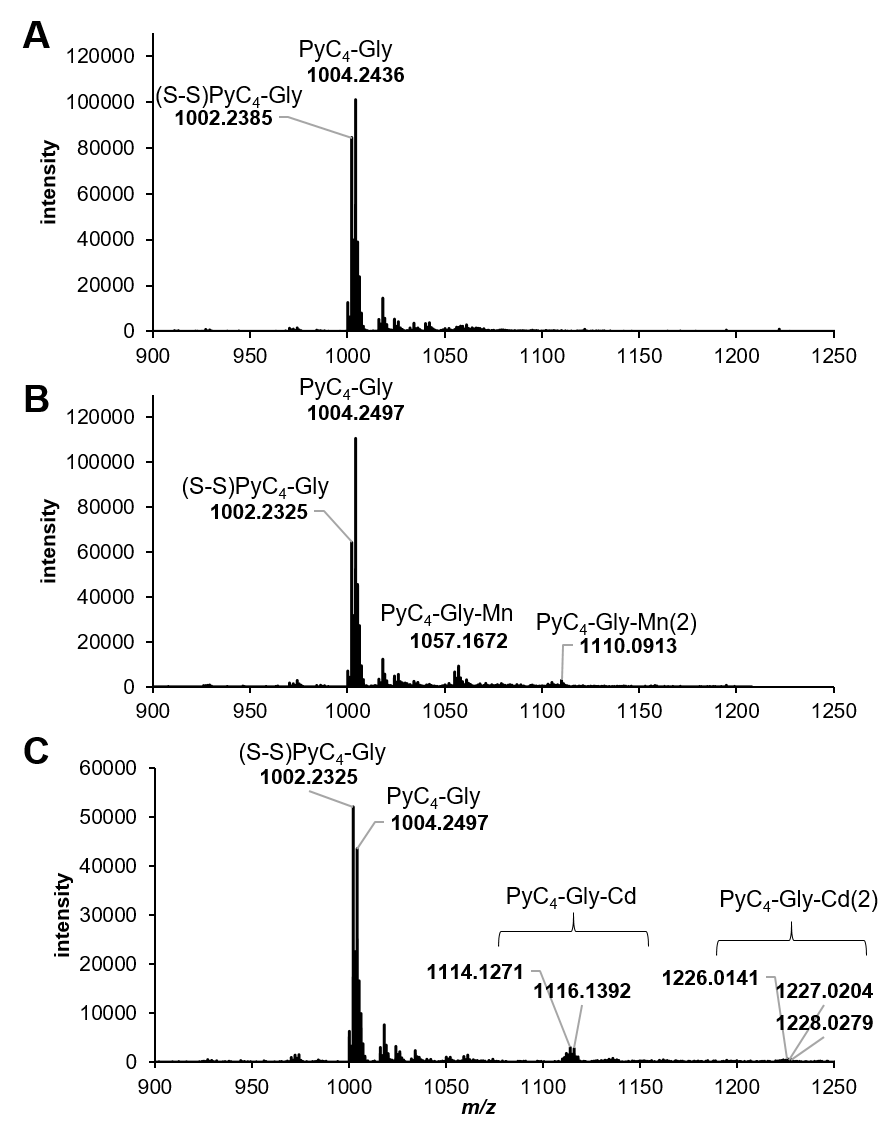


**Figures S-7.** MS1 spectra of PyC_4_-Gly authentic standard alone (**A**) and in combination with equimolar MnCl_2_ (**B**) or CdCl_2_ (**C**). (S-S)PyC_4_-Gly, oxidized form of PyC_4_-Gly; PyC_4_-Gly, reduced form of PyC_4_-Gly; PyC_4_-Gly-Mn, PyC_4_-Gly complexed with Mn^2+^ ion; PyC_4_-Gly-Cd, PyC_4_-Gly complexed with Cd^2+^ ion; PyC_4_-Gly-Cd(2), PyC_4_-Gly complexed with two Cd^2+^ ions.


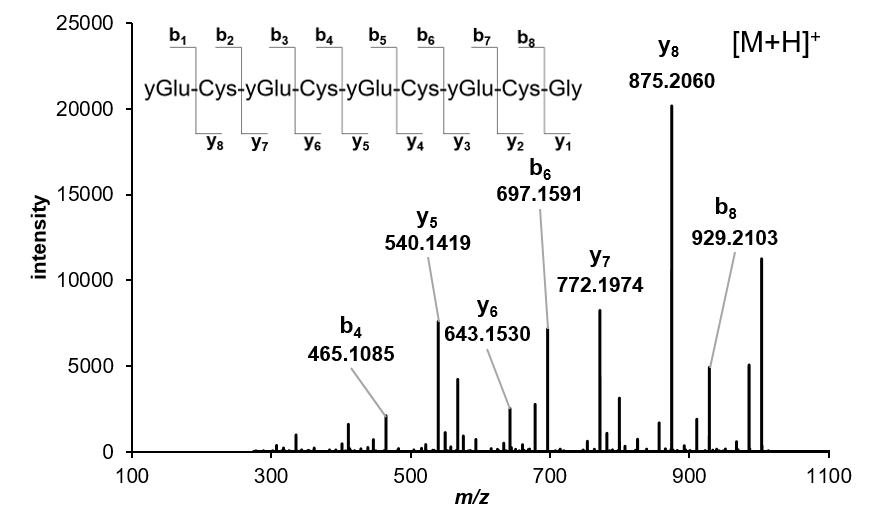


**Figure S-8. MS/MS of PyC_4_-Gly.** Fragmentation pattern for PyC_4_-Gly [M+H] (*m/z* 1004.25) from the authentic standard sample using collision-induced dissociation (CID) at 25V.

**References**

1. Go, Y.M., Uppal, K., Walker, D.I.*, et al.* (2014) Mitochondrial metabolomics using high-resolution Fourier-transform mass spectrometry. *Methods Mol Biol*, **1198**, 43-73.

2. Soltow, Q.A., Strobel, F.H., Mansfield, K.G.*, et al.* (2013) High-performance metabolic profiling with dual chromatography-Fourier-transform mass spectrometry (DC-FTMS) for study of the exposome. *Metabolomics*, **9**, S132-S143.

3. Yu, T., Park, Y., Johnson, J.M.*, et al.* (2009) apLCMS--adaptive processing of high-resolution LC/MS data. *Bioinformatics*, **25**, 1930-1936.

4. Uppal, K., Soltow, Q.A., Strobel, F.H.*, et al.* (2013) xMSanalyzer: automated pipeline for improved feature detection and downstream analysis of large-scale, non-targeted metabolomics data. *BMC Bioinformatics*, **14**, 15.

5. Cobbett, C., Goldsbrough, P. (2002) Phytochelatins and metallothioneins: roles in heavy metal detoxification and homeostasis. *Annu Rev Plant Biol*, **53**, 159-182.

6. Serrano, N., Diaz-Cruz, J.M., Arino, C.*, et al.* (2015) Recent contributions to the study of phytochelatins with an analytical approach. *Trends in Analytical Chemistry*, **73**, 129-145.

7. Uppal, K., Walker, D.I., Jones, D.P. (2017) xMSannotator: An R Package for Network-Based Annotation of High-Resolution Metabolomics Data. *Anal Chem*, **89**, 1063-1067.

8. Schymanski, E.L., Jeon, J., Gulde, R.*, et al.* (2014) Identifying small molecules via high resolution mass spectrometry: communicating confidence. *Environ Sci Technol*, **48**, 2097-2098.

9. Wolf, S., Schmidt, S., Muller-Hannemann, M.*, et al.* (2010) In silico fragmentation for computer assisted identification of metabolite mass spectra. *BMC Bioinformatics*, **11**, 148.

10. Ruttkies, C., Schymanski, E.L., Wolf, S.*, et al.* (2016) MetFrag relaunched: incorporating strategies beyond in silico fragmentation. *J Cheminform*, **8**, 3.

11. Kim, S., Thiessen, P.A., Bolton, E.E.*, et al.* (2016) PubChem Substance and Compound databases. *Nucleic Acids Res*, **44**, D1202-1213.
